# A-site deficient semiconductor electrolyte Sr_1−*x*_Co_*x*_FeO_3−*δ*_ for low-temperature (450–550 °C) solid oxide fuel cells†

**DOI:** 10.1039/d2ra03823d

**Published:** 2022-08-30

**Authors:** Yuzheng Lu, M. A. K. Yousaf Shah, Naveed Mushtaq, Muhammad Yousaf, Peter D. Lund, Bin Zhu, Muhammad Imran Asghar

**Affiliations:** School of Electronic Engineering, Nanjing Xiao Zhuang University 211171 Nanjing China; Jiangsu Provincial Key Laboratory of Solar Energy Science and Technology/Energy Storage Joint Research Center, School of Energy and Environment, Southeast University No. 2 Si Pai Lou Nanjing 210096 China alikamranshah91@gmail.com; New Energy Technologies Group, Department of Applied Physics, Aalto University School of Science P. O. Box 15100, FI-00076 Aalto Espoo Finland imran.asghar@aalto.fi; Faculty of Physics and Electronic Science, Hubei University Wuhan Hubei 430062 China

## Abstract

Fast ionic conduction at low operating temperatures is a key factor for the high electrochemical performance of solid oxide fuel cells (SOFCs). Here an A-site deficient semiconductor electrolyte Sr_1−*x*_Co_*x*_FeO_3−*δ*_ is proposed for low-temperature solid oxide fuel cells (LT-SOFCs). A fuel cell with a structure of Ni/NCAL-Sr_0.7_Co_0.3_FeO_3−*δ*_–NCAL/Ni reached a promising performance of 771 mW cm^−2^ at 550 °C. Moreover, appropriate doping of cobalt at the A-site resulted in enhanced charge carrier transportation yielding an ionic conductivity of >0.1 S cm^−1^ at 550 °C. A high OCV of 1.05 V confirmed that neither short-circuiting nor power loss occurred during the operation of the prepared SOFC device. A modified composition of Sr_0.5_Co_0.5_FeO_3−*δ*_ and Sr_0.3_Co_0.7_FeO_3−*δ*_ also reached good fuel cell performance of 542 and 345 mW cm^−2^, respectively. The energy bandgap analysis confirmed optimal cobalt doping into the A-site of the prepared perovskite structure improved the charge transportation effect. Moreover, XPS spectra showed how the Co-doping into the A-site enhanced O-vacancies, which improve the transport of oxide ions. The present work shows that Sr_0.7_Co_0.3_FeO_3−*δ*_ is a promising electrolyte for LT-SOFCs. Its performance can be boosted with Co-doping to tune the energy band structure.

## Abstract 2

Fast ionic conduction at low operating temperatures is a key factor for the high electrochemical performance of solid oxide fuel cells (SOFCs).

## Graphical abstract

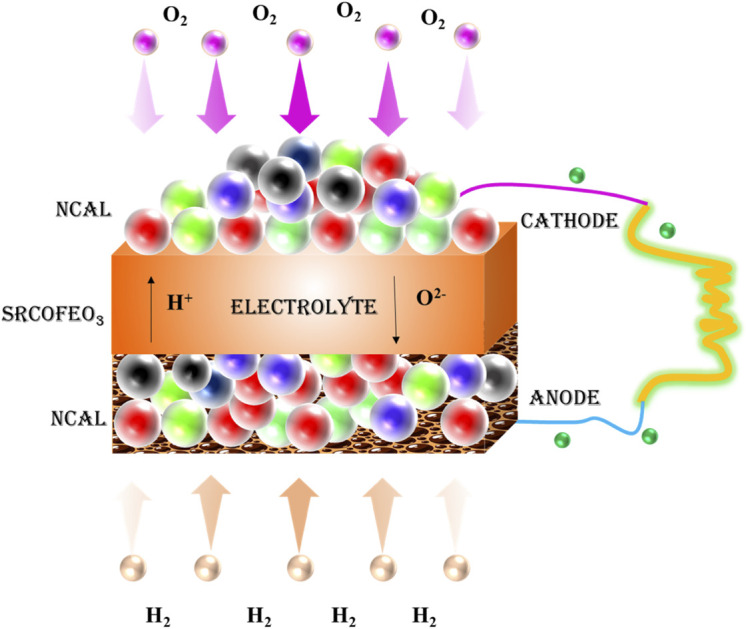

## 1 Introduction

The intermediate and lower temperature solid oxide fuel cells (IT-SOFC & LT-SOFCs) are an interesting alternative to the high-temperature solid oxide fuel cell (HT-SOFC). The challenge with these alternatives is to design suitable electrolytes which deliver high enough ionic conductivity at lower temperatures.^1,2^ The traditional YSZ (8% Y_2_O_3_ stabilized ZrO_2_) for SOFC is a reliable and stable electrolyte providing a maximum ionic conductivity of 0.1 S cm^−1^ at 800–1000 °C. However, the high operating temperature has many drawbacks such as fast degradation, and slow start-up and shut-down cycle which hamper the commercialization of SOFC technology.^1–3^ In contrast, the low-temperature SOFC has issues with low ionic conductivity and higher losses (ohmic losses).^2^ The introduction of new materials as alternative electrolytes (GDC, GDC/YSZ) and fabrication of ultra-thin electrolytes (adopted by thin-film technologies) (YSZ/GDC) with small ohmic resistance has often been presented as solutions to the above-stated challenges.^4–9^ However, the high cost and scaling-up difficulties in the adaptation of thin-film technology have a detrimental impact on the long-term stability of the fuel cell stack.^10^ As a result, much work has gone into developing novel materials that could overcome the challenges mentioned above while also providing high ionic conductivity (>0.1 S cm^−1^) in the low-temperature range (<600 °C).^2^

Novel approaches such as semiconductor membrane (SM) and semiconductor ionic membrane (SIM) based materials have been introduced as electrolytes. Semiconductor materials possess both ionic and electronic conductivity, which is beneficial for higher OCV and better fuel cell performance, especially at low operational temperatures. An important benefit of semiconducting materials is the high ionic conductivity without short-circuiting issues.^11,12^ Also, metal oxides, perovskites, fluorite, and layered structure such as TiO_2_, ZnO, CeO_2,_ and NCAL (Ni_0.8_Co_0.15_Al_0.05_LiO_2_) falls under the semiconducting materials with enhanced electrical properties.^13–16^ Semiconductor ionic membranes have played an important role in improving the performance of fuel cell devices.^12^ High performance is due to the heterojunction between the semiconductor and ionic conductor, which further forms a space charge region constituting BIEF (built-in electric field).^17^ For example, SFT–SDC (SrFe_0.75_Ti_0.25_O_3−*δ*_–Sm_0.25_Ce_0.75_O_2−*δ*_) is an efficient electrolyte delivering high fuel cell performance and high ionic conductivity, which can be explained through the formation of a heterojunction between the semiconductor SFT and ionic conductor SDC.^17^ Moreover, Xia *et al.* have reported BCFZY–ZnO as a p–n junction to be used as an electrolyte to deliver higher fuel cell performance and ionic conductivity without any electron short-circuiting, especially at low operating temperatures of 450–550 °C.^18^ A range of other SIM materials for fuel cell technology has been reported.^19–21^

Many single-phase semiconductor materials, especially the perovskite and layered structures are alternative candidates for SOFC technology despite the heterostructure associated with them. For example, the perovskite structure (SmNiO) was used as an electrolyte in a fuel cell yielding a power density of 225 mW cm^−2^ and a high OCV of 1.02 V at a low operating temperature of 500 °C.^22^ Moreover, Co-doped SrSnO_3_ was used recently as a semiconductor electrolyte to reach 497 mW cm^−2^ and high ionic conductivity of 0.12 S cm^−1^ at 520 °C.^23^ Fe-doped SrTiO_3_ as an electrolyte has also reached fuel cell performance and ionic conductivity at 520 °C.^24^ Chen *et al.* synthesized a semiconductor perovskite SrTiO_3_ and applied it as electrolyte which reached a high-power density of 600 mW cm^−2^ at 550 °C.^25^ The layered structure Li_*x*_Co_0.5_Al_0.5_O_2_ (LCAO) was synthesized in ref. 26 and inserted as an electrolyte with the following configuration of Ag/LCAO/Ag, yielding 180 mW cm^−2^ at 525 °C Here we employ SrCoFeO_3_ (SCF) as an electrolyte to SOFC. It has perovskite and semiconductor characteristics with very good electrical multifunctional properties, but to our best knowledge, it has not been applied before to a fuel cell.

We have synthesized SrCoFeO_3_ to be employed as an electrolyte in between symmetrical Ni–NCAL electrodes with a sandwich configuration of Ni–NCAL/SCF/NCAL-Ni for fuel cell operation. Electrochemical and detailed characterization analyses such as XRD, SEM, HR-TEM, XPS, and UV-visible spectroscopy were performed in detail. Obtained electrical and electrochemical properties were compared with previously published results on semiconductor perovskite electrolytes. Also, the influence of doping on the energy bandgap and its effects on the fuel cell performance and ionic conductivity were investigated.

## 2 Materials and methods

### 2.1. Material synthesis

The sol–gel method was used to synthesize the SrCoFeO_3_ powder as an electrolyte for the fuel cell. In detail, at first, the 200 mL water was poured into a beaker for stirring, then the appropriate amount of all materials such as Sr(NO_3_)_3_·6H_2_O, Co(NO_3_)_3_·6H_2_O and Fe(NO_3_)_3_·6H_2_O (Sigma Aldrich 99.9% purity) was mixed in sequence and left for 30 min for proper and uniform mixing. After 30 min of adequate mixing, the appropriate amount of citric acid was added to work as a chelating agent or to chelate the metal nitrates and keep stirring and heating at 80 °C with 5° min^−1^ and 500 rpm rate till the brownish gel is formed. The metal cations to citric acid ratio were 1 : 1.

Consequently, the temperature of the hot plate was increased to 110 °C to alter the gel into ashes. The resulted ashes were grounded adequately and sintered at 800 °C for 4 hours to obtain the fine powder of SrCoFeO_3_. Similarly, the other composition, Sr_0.5_Co_0.5_FeO_3_, and Sr_0.5_Co_0.7_FeO_3_ was also prepared in the identical way as Sr_0.7_Co_0.3_FeO_3_.

### 2.2. Material characterization

To investigate the structural phase of Sr_1−*x*_Co_*x*_FeO_3_ (*x* = 0.3–0.7), the X-ray diffraction (XRD) was executed through an X-ray diffractometer where Cu (K-alpha) was used as a source of radiation. But FE-SEM (Field emission-scanning electron microscopy) was deployed to examine the morphology of synthesized powder of Sr_1−*x*_Co_*x*_FeO_3_. Also, a detailed inspection of prepared Sr_0.7_Co_0.3_FeO_3_ was performed using (HR-TEM, Tecnai, G2 F30) high resolution-transmission electron microscopy. The accelerating voltage was kept at 300 kV. X-ray photoelectron spectroscopy (XPS) analysis was used to study the chemical and surface properties (oxidation state or chemical species of surface atoms). The UV-visible (UV-3600 spectrometer, MIOSTECHPTY Ltd) was utilized to determine the energy bandgap (*E*_g_) of Sr_1−*x*_Co_*x*_FeO_3_.

### 2.3. Assembly and measurements of the pellet

After synthesizing the final powder, the first step is to prepare the electrodes Ni–NCAL, in which Ni foam was circled into the pellet shape of 13 mm diameter. At the same time, the 2^nd^ steps were to make the delicate and dense slurry of NCAL (Ni_0.8_Co_0.15_Al_0.05_LiO_2_) powder by mixing the appropriate amount of NCAL and terpinol binder. The ration of binder to NCAL was 1%. The used NCAL was purchased from China Bamo Tec joint-stock Ltd company. Afterward, the 3^rd^ step is mixing the slurry uniformly and then brushing it on the Ni-foam to gain the Ni–NCAL electrodes; later, the prepared Ni-pasted NCAL electrodes were dried for almost 30 min at the temperature of 80°. The fourth step is to censor the prepared circular-shaped Ni–NCAL electrodes with a 13 mm diameter. The 5^th^ step is to take 0.3 grams of prepared electrolyte powder and two symmetrical electrodes (Ni–NCAL). The electrolyte is sandwiched among the two symmetrical electrodes and then compressed under an applied pressure of 360 MPa to attain a fine pellet of 0.64 cm^2^ in area and 13 mm in diameter.

Moreover, the thickness of the pellet was kept at 1.5 mm, where electrolyte thickness was about 750 μm. The final step was to take the pellet and fix it in a sample holder testing device to test the prepared materials' electrical and electrochemical performance. Another composition of electrolyte materials, Sr_0.5_Co_0.5_FeO_3_ and Sr_0.3_Co_0.7_FeO_3_, was used to prepare two more pellets in the same manner for comparison purposes.

Afterward testing holder was placed in a furnace to heat the pellet. After 1 hour, the hydrogen fuel (at 80–120 ml min^−1^ of flow rate) and air (at 150–200 ml min^−1^) were supplied to the testing device to realize the fuel cells' functionality. Later, the electronic load (IT8511, ITECH Electrical Co., Ltd) was run to test the current–voltage (*I*–*V*) and current–power (*I*–*P*) characteristics curves for the synthesized materials. Moreover, Gammry-reference 3000 USA device was deployed to run the electrochemical Impedance (EIS) spectra, which were fixed to determine the electrochemical properties under H_2_/air environment. The deployed EIS spectra frequency and amplitude were set in the given range (from 0.1 Hz to 1 MHz) and amplitude (10 mV), respectively.

## 3 Results and discussion

**Fig. 1:**
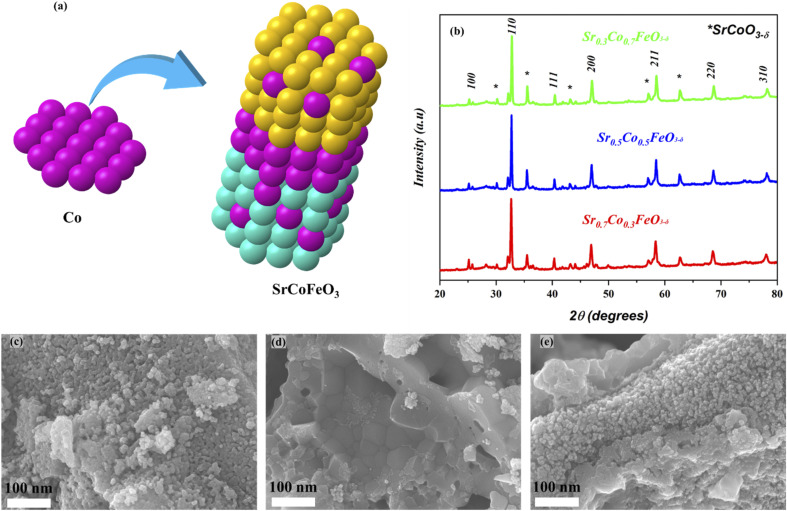
(a and b) Crystal structure and XRD result of synthesized lattice Sr_1−*x*_Co_*x*_FeO_3−*δ*_ sintered at 800 °C while (c–e) the FE-SEM images of Sr_1−*x*_Co_*x*_FeO_3_ (*x* = 0.3–0.7) respectively.

The XRD analysis of prepared powder of perovskite SrCoFeO_3_ structure with different compositions has been presented in Fig. 1(b). Also, the Co is doped to A site of prepared perovskite structure Sr_1−*x*_Co_*x*_FeO_3,_ as can be confirmed in Fig. 1(a). Moreover, the characteristics peaks (100), (110), (111), (200), (211), (220), and (310) of Sr_1−*x*_Co_*x*_FeO_3_ evidence the PVK structure of the cubic phase with the space group of *Pm̄*3*m* and reference# 01-082-2445.^24^ Also, there are minor peaks of SrCoO_3_ appearing in the prepared structure, signifying another phase (hexagonal structure), which might be due to the access amount of cobalt doping into Sr.^27^ The other aspect is using a low sintering temperature to calcined the prepared powder. These two factors might be the solid reason for the impurity peaks in the prepared structure. Also, the peaks are shifting towards a higher angle as cobalt content increases into the A-site of prepared perovskite due to the difference in atomic radii of Sr (2.51 Å) and Co (1.52 Å) following the Vegard's law.^23,24^ Also, the difference in atomic radii leads to higher content of O-vacancies in the prepared structure. Moreover, the prepared structure and characteristics peaks are according to the previously published literature. The cobalt was doped into SrFeO_3_ to obtain the SrCoFeO_3_ structure, as displayed in Fig. 1(a).

Fig. 1(c–e) reveals the morphology of the prepared structure Sr_1−*x*_Co_*x*_FeO_3_ (*x* = 0.3–0.7) in nano-scale range (100 nm). The FE-SEM image confirms that cobalt doping alters the surface morphology into the uniform distribution of rod shape particles, as displayed in Fig. 1(c–e). Also, the appropriate amount of Co doping into SrFeO_3_ enhances the concentration of particles. It increases the active sites of prepared materials, which benefits the charge transportation of prepared symmetry. Also, the obtained morphology reveals that all particles are well connected and compacted coherently, which significantly benefits the electrochemical performance and charge transportation. Also, the SEM image of Ni foam and SEM cross-section of the pellet have been shown in ESI Fig. S1(a–c).†

**Fig. 2:**
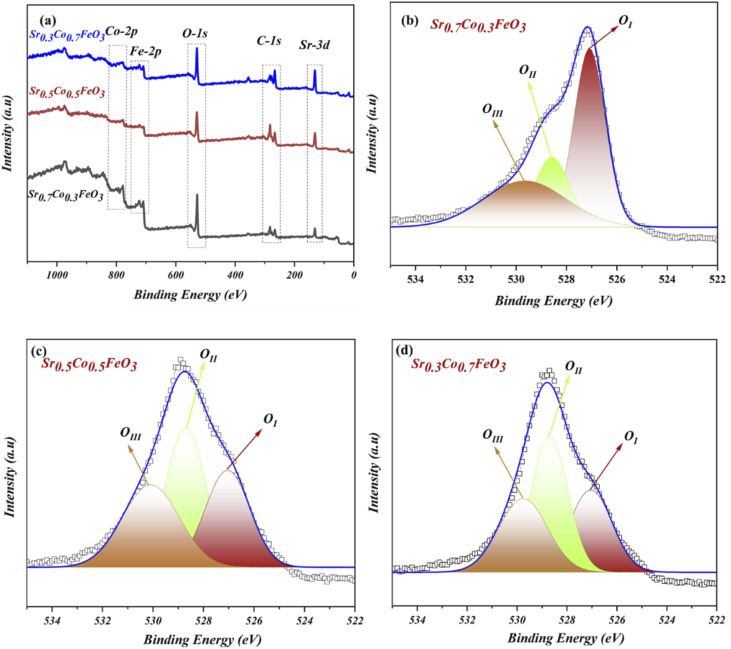
(a) Full XPS (X-ray photoelectron spectroscopy) spectra of prepared SrCoFeO_3_, (b–d) O-1s spectra of Sr_1−*x*_Co_*x*_FeO_3_ (*x* = 0.3–0.7).

The XPS (X-ray photoelectrochemical spectroscopy) was performed to examine the chemical composition and oxidation states to study the surface properties of the respective composition of Sr_1−*x*_Co_*x*_FeO_3_ (*x* = 0.3–0.7). According to the previous reports, the XPS data were fitted using the origin Software, where C-1s spectra were set as reference points to correct the spectra of all elements (Sr, Co, Fe and O). The complete XPS spectra of prepared material Sr_1−*x*_Co_*x*_FeO_3−*δ*_ have been displayed in Fig. 2(a), confirming the presence of each element, such as Sr-3d, Co-2p, Fe-2p, and O-1s. The XPS spectra of Sr-3d, Co-2p, and Fe-2p have been presented in the ESI Fig. S2(a–c).†

The O-1s spectra of prepared composition Sr_1−*x*_Co_*x*_FeO_3_ (*x* = 0.3–0.7) have been presented in Fig. 2(b–d). The three deconvoluted peaks (O_I_, O_II,_ and O_III_) of oxygen spectra of Sr_1−*x*_Co_*x*_FeO_3_ (*x* = 0.3–0.7) have been assigned to different binding energies. Such as the existing and deconvoluted peaks located at 527 eV and 529.5 eV, correlated to the lattice oxygen and surface adsorbed oxygen. These peaks are denoted as O_I_ and O_II_ in the XPS spectra of O-1s, as shown in Fig. 2(b–d). Also, the peak area has been modified by appropriate cobalt doping into the A-site of prepared perovskite Sr_0.7_Co_0.3_FeO_3_.^13,14,28,29^

In contrast, cobalt doping enhances the area of the peak that got shrinks which claims that appropriate doping of cobalt might benefit the structural changes and charges transfer in prepared Sr_1−*x*_Co_*x*_FeO_3_. The enhancement in the peak area is due to merging more defects in the lattice and enhancing the lattice parameter, leading to more active sites, which overall causes the ionic conduction—the peak located at 531.3 eV cross-ponds to the O-vacancies or surface oxide defects. Higher binding energy signifies the presence of O-vacancies, which mainly benefits the charge transport in the prepared lattice.^29^ Furthermore, the peaks between 528 and 532 eV manifest the presence of oxide ions and oxygen vacancies, which is crucial for high ionic conduction and electrochemical fuel cell performance.^13,14^

**Fig. 3:**
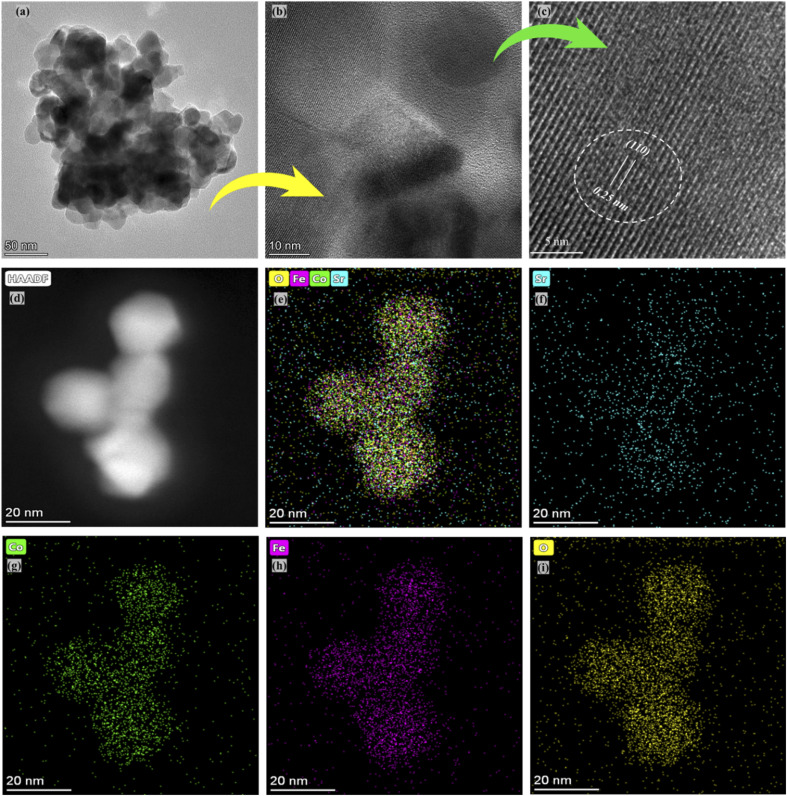
(a–c) HR-TEM images of Sr_0.7_Co_0.3_FeO_3_ and (d–i) selected images for mapping and EDS mapping of all elements, including Sr, Co, Fe, and O.

The selected composition or the optimal composition Sr_0.7_Co_0.3_FeO_3_ was assigned to characterize the morphology further using HR-TEM assisted with mapping all elements as shown in Fig. 3. Also, HR-TEM revealed the buried surface and interface grain boundaries among the particles, which mainly influence the origin and enhancement of ionic conduction of Sr_0.7_Co_0.3_FeO_3_. Furthermore, the HR-TEM images revealed the uniform packing and viscous adhesion among the particles led to the densification of the prepared lattice. Also, such constructed network and interaction or strong bondage among the particles causes enhances the ionic conduction through the interface grain boundary, as shown in Fig. 3(a and b).^30^Fig. 3(c) displayed the calculated d-spacing value of 0.25 nm correlated to the lattice fringes (110) of synthesized powder of Sr_0.7_Co_0.3_FeO_3_.^31^ Moreover, the selected and suitable morphology was chosen for mapping all elements (Sr, Co, Fe, and O) in the synthesized composition Sr_0.7_Co_0.3_FeO_3_, as displayed in Fig. 3(d–i). Also, EDS images confirmed the successful and uniform incorporation to the A-site of Sr_0.7_Co_0.3_FeO_3_ lattice.

**Fig. 4:**
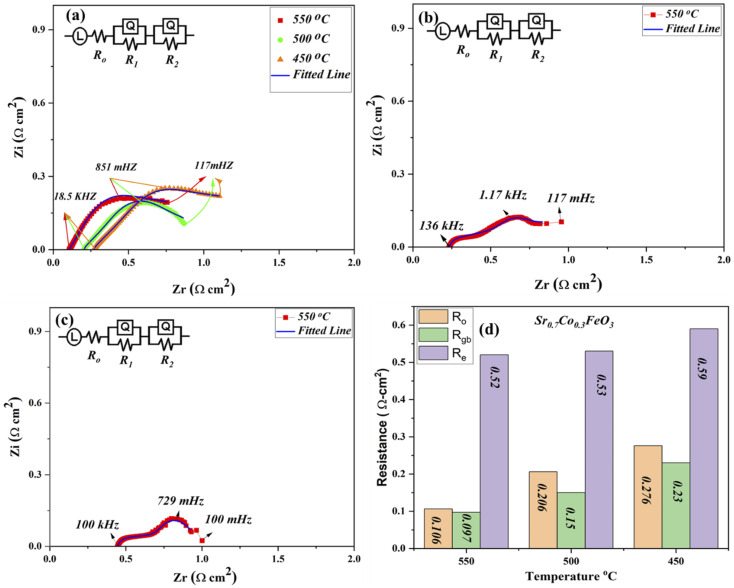
(a) EIS spectra of Sr_0.7_Co_0.3_FeO_3_ under H_2_/air at 550–450 °C, (b and c) EIS spectra of other compositions Sr_0.7_Co_0.5_FeO_3_ and Sr_0.7_Co_0.7_FeO_3_ under H_2_/air at 550 °C (d) comparison of EIS resistance values of Sr_0.7_Co_0.3_FeO_3_ at 550–450 °C.

The EIS (electrochemical impedance spectroscopy) was used to evaluate the electrical characteristics of prepared materials Sr_0.7_Co_0.3_FeO_3_ under the open-circuit voltage condition in H_2_/air environment at low operational temperature 550–450 °C as depicted in Fig. 4(a).^29,30^ Other composition Sr_1−*x*_Co_*x*_FeO_3_ (*x* = 0.5–0.7) data have been operated at 550 °C as displayed in Fig. 4(b and c). The full EIS spectra have been displayed in ESI (Fig. S3†). The ohmic, grain boundary, and electrode resistance are evaluated *via* fitting the obtained EIS curve using Z-simpwin Software. Also, the ohmic and polarization resistance were studied through the variation in frequency. The starting point of intersection at the real axis corresponds to the ohmic resistance, mainly electrolyte and electrode resistance. The high frequency, intermediate frequency, and lower frequency regions are cross ponds to the ohmic, grain boundary, and electrode resistance, as pointed out in Fig. 4. The Z-simpwin Software was designed to fit the data by employing the fitted circuit *LR*_o_(*R*_1_*Q*_1_) (*R*_2_*Q*_2_), where *R*_o_ is the ohmic resistance, *R*_1_ is the grain boundary resistance, and *R*_2_ is the electrode resistance while *Q* is constant phase elements which can be considered as a no-ideal capacitance.^23,24,29,30^

The exact resistance values have been compared in Fig. 4(d), especially at 550 °C under H_2_/air environment. Also, *R*_1_ and *R*_2_ are known as the polarization resistance, where *R*_1_ is the resistance between the electrolyte and electrode to transport ions, while *R*_2_ manifests the adsorption and dissociation of oxide ions in the prepared lattice. The obtained results show that both ohmic and grain boundary resistance reduced, leading to high oxygen vacancies and charge transport thermally, especially at the grain boundary in the Sr_1−*x*_Co_*x*_FeO_3_ (*x* = 0.3–0.7) electrolyte layer. Also, ohmic and polarization resistance of Sr_0.7_Co_0.3_FeO_3_ is lower than the other prepared composition at 550 °C, manifesting that optimal doping is the solid reason to reduce the resistance of either ohmic or polarization resistance, which majorly caused to enhance the charge transportation and fuel cell performance. Moreover, the ohmic and grain boundary resistance and the electrode resistance got reduced, signifying the better electrode activity of prepared Sr_0.7_Co_0.3_FeO_3_. The EIS results manifest that Sr_0.7_Co_0.3_FeO_3_ might be suitable materials for fuel cell technology.

**Fig. 5:**
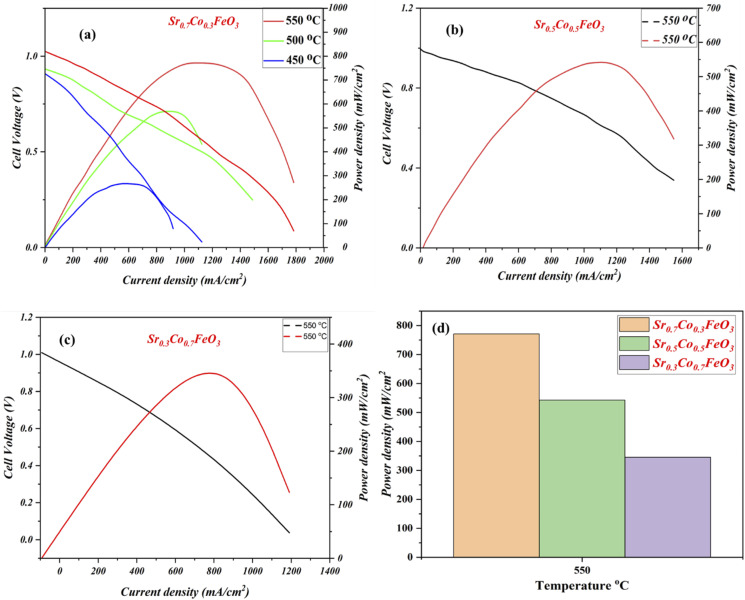
(a–c) Fuel cell performance of Sr_0.7_Co_0.3_FeO_3_ under H_2_/air at 550–450 °C, (d) comparison of Fuel cell performance of Sr_1−*x*_Co_*x*_FeO_3_ (*x* = 0.3–0.7) at 550 °C.

The electrochemical fuel cell performance in terms of *I*–*V*/*I*–*P* characteristics curves of synthesized electrolyte material Sr_0.7_Co_0.3_FeO_3_ has been performed under the H_2_/air environment at different operating temperatures 550–450 °C as shown in Fig. 5(a). The fuel cell performance mainly depends on the content of cobalt doping, as depicted in Fig. 5(a–c). The appropriate doping of cobalt like 30% (Sr_0.7_Co_0.3_FeO_3_) can enhance the fuel cell performance from 250 mW cm^−2^ to 771 mW cm^−2^ in different temperatures 450–550 °C. Also, the appropriate doping claimed a higher OCV of 1.03 V at 550 °C, claiming neither degradation nor the short-circuiting issue happened during the fuel cell performance.^23,24^ In contrast, other compositions have been tested compared to the Sr_0.7_Co_0.3_FeO_3_ and delivered meaningful fuel cell performance (542 & 342 mW cm^−2^) but not higher than the Sr_0.7_Co_0.3_FeO_3_ 771 mW cm^−2^ at 550 °C manifesting that appropriate doping of cobalt leads to higher fuel cell performance as can be seen in Fig. 5(d). So, the doping and thermal effect significantly impact the fuel cell performance and enhance ionic transport in an electrolyte, as clarified in Fig. 5(a–c). Moreover, XRD results have claimed that the difference in ionic radius of Sr and Co causes more disorder in structure, leading to defects then O-vacancies, which overall enhance the ionic conductivity of prepared Sr_0.7_Co_0.3_FeO_3_ electrolyte material for a fuel cell. Besides, the obtained fuel cell performance is higher than the reported (YSZ, SD, BCZYY) literature under identical operating condition manifesting the credible electrolyte for LT-SOFC.^32–34^

**Fig. 6:**
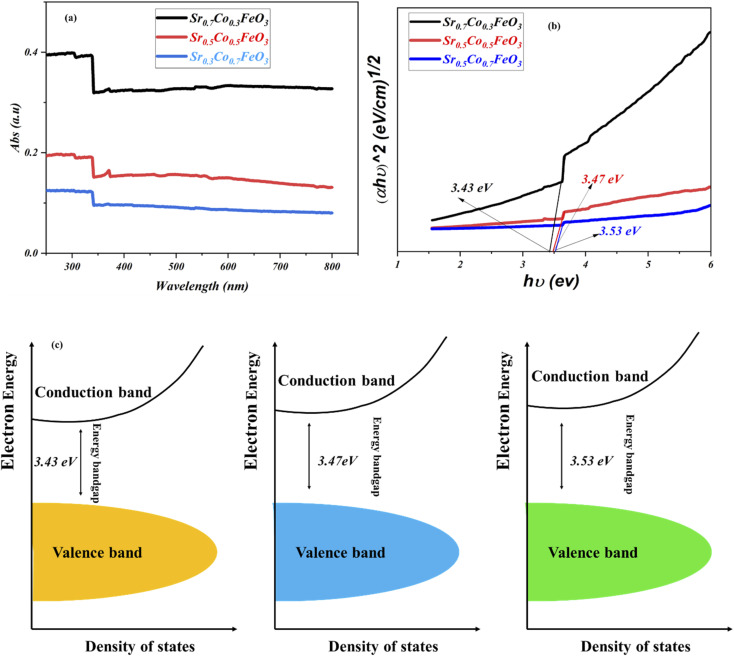
(a and b) UV-visible spectra of Sr_1−*x*_Co_*x*_FeO_3_ (*x* = 0.3–0.7), (c) energy bandgap diagram of all composition of comparison of Sr_1−*x*_Co_*x*_FeO_3_ (*x* = 0.3–0.7).

Furthermore, it is essential to design materials with benefitted results in terms of electrical and electrochemical, which is only possible *via* investigation of energy bandgap. As energy bandgap plays a crucial role in triggering the material's functionality, especially for the fuel cell application, which has been ignored in the past. To investigate the doping effect on the energy bandgap and on the fuel cell performance and charge transport, the UV-visible spectra were performed to evaluate the energy bandgap of Sr_1−*x*_Co_*x*_FeO_3_ (*x* = 0.3–0.7), as shown in Fig. 6. The absorbance spectra clued the energy bandgap among the conduction and valence. Also, the definite energy needed for the electron movement from the valence band to the conduction band can be regarded as the energy bandgap. The absorption spectra revealed that the appropriate doping like 30% is the maximum absorption edge, while as doping increases from the 30 to 70%, the absorption shift towards the lower wavelength signifying the increment in the bandgap due to higher content of cobalt doping (depicted in Fig. 6(a and b)).^23,24^ The following equation *αhv* = *A*(*hv* − *E*_g_)^*n*^ gives the calculated energy bandgap values such as 3.53 to 3.43 eV cross ponds to the Sr_1−*x*_Co_*x*_FeO_3_ (*x* = 0.7–0.3). The appropriate doping of cobalt might be responsible for increasing the electron–hole concentration and expanding bondage length, enhancing the carrier concentration and fuel cell performance.^23^Fig. 6(c) reveals the energy bandgap diagram confirming that appropriate doping causes to reduce the bandgap. Still, excess doping causes to increase the bandgap. The energy bandgap results have claimed that appropriate doping is in favor of reducing the energy bandgap also better charge transportation between valence and conduction band, while excess doping leads to enhancing the energy bandgap also might create an excess number of defects which causes to decline in charge transportation and finally reduce the performance of the device as confirmed in above-stated results.^35^

**Fig. 7:**
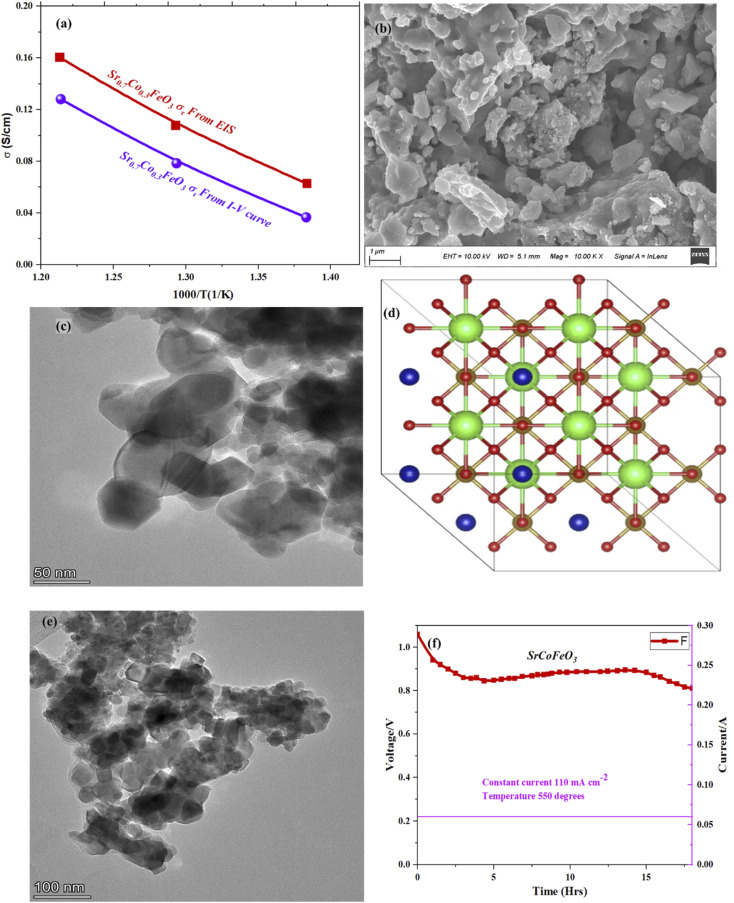
(a) The total and ionic conductivity of synthesized electrolyte Sr_0.7_Co_0.3_FeO_3_ at different temperatures 550–450 °C, (b, c, e) SEM and HR-TEM morphology of Sr_0.7_Co_0.3_FeO_3_ while (d) the side view of the crystal structure of Sr_0.7_Co_0.3_FeO_3_ with 101 planes, (f) durability of Sr_0.7_Co_0.3_FeO_3_ at 550 °C.

Furthermore, the electrical conductivity of SrCoFeO_3_ have been evaluated at different operational temperature 550–450 °C as depicted in Fig. 7(a). The total conductivity of prepared electrolyte has been computed using the EIS analysis while the ionic conductivity is determined by employing the Ohm’s law through accomplished *I*–*V* curve of fuel cell performance. The linear and central part of the *I*–*V* curve can be considered as the outcome of the ohmic resistance of electrolyte and electrode and the selected portion of the *I*–*V* curve is associated to the total ohmic polarization losses.^18^ Also, the total ohmic resistance of the *I*–*V* curve is probably equivalent to the ionic resistance of SrCoFeO_3_ because the electronic resistance offered by NCAL/Ni is negligible in comparison of ionic resistance of electrolyte. The resistance of electrolyte can be determined by using the Ohm law as stated below;^30^
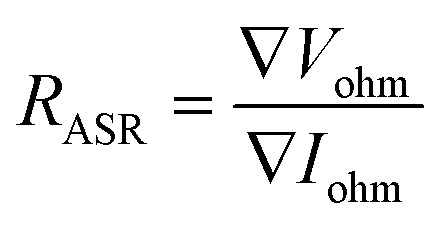
where the ∇*V*_ohm_ the ohmic polarization losses along with decline current ∇*I*_ohm_. With the help of ohmic resistance the ionic conductivity of electrolyte is calculated using the following relation.
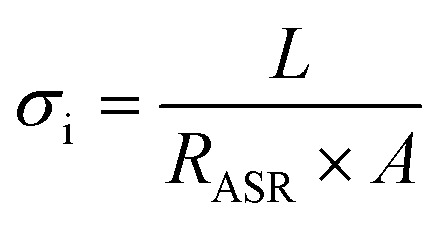


In the above equation, the *L* is the thickness of the electrolyte *R* is the calculated ohmic resistance of the electrolyte and *A* is the cross-sectional of the assembled pellet. The obtained ionic conductivity of SrCoFeO_3_ from the *I*–*V* curve is 0.13 S cm^−1^ out of the total conductivity of 0.16 S cm^−1^ calculated from the EIS revealing the dominant ionic conduction in the synthesized SrCoFeO_3_ as shown in Fig. 7(a). The obtained ionic conductivity is higher than the reported literature suggesting the prepared electrolyte is a capable electrolyte for fuel cell application.^32–34^Fig. 7(b and c) shows the morphology images of SEM and HR-TEM revealing that particles are well connected which might produce continuous channels for ions transportation with low activation energy. The Fig. 7(d) shows the crystal structure of SrCoFeO_3_ perovskite with a direction (110) plane. All the above results have proven that SrCoFeO_3_ is a competent and most capable material for electrolyte application in fuel cells, especially in the range of low temperatures. Short-term durability of about 16 hours with the constant current density of 110 mA cm^−2^ was performed at 550 °C as shown in Fig. 7(f). The OCV droppages might be observed due to the densification issue gas cross over or chemical reaction. Furthermore, a new test setup with advanced engineering technology is compulsory for long term stability.

## 4 Conclusion

In summary, we have synthesized Sr_0.7_Co_0.3_FeO_3_ and used it as an electrolyte in a fuel cell at different temperatures. The fuel cell performance with Sr_0.7_Co_0.3_FeO_3_ was very good: a power density 771 mW cm^−2^ at 550 °C was reached, the ionic conductivity was 0.13 S cm^−1^ including a high OCV of 1.03 V. The XRD, SEM and HR-TEM results indicated that Sr_0.7_Co_0.3_FeO_3_ has a cubic structure and fine morphology with homogeneously distributed particles in the nanoscale. The XPS result showed that appropriate doping of cobalt produced more O-vacancies which increased the ionic conductivity of the electrolyte layer. The Sr_0.7_Co_0.3_FeO_3_ obtained a high ionic conductivity of 0.13 S cm^−1^ at 550 °C. Also, the energy bandgap played a crucial role in enhancing charge carrier transport. The results indicate that Sr_0.7_Co_0.3_FeO_3_ is a potential candidate for electrolyte applications for fuel cells.

## Conflicts of interest

We do not have any competing financial interests or personal relationships that can influence the current work's reported paper.

## Supplementary Material

RA-012-D2RA03823D-s001
