# Supplementary material for: A-site deficient semiconductor electrolyte Sr1−xCoxFeO3−δ for low-temperature (450–550 °C) solid oxide fuel cells
Source: RSC Adv. 2022 Aug 30;12(38):24480–90. doi: 10.1039/d2ra03823d (PMC9426435; doi:10.1039/d2ra03823d)
Supplement: RA-012-D2RA03823D-s001 [file RA-012-D2RA03823D-s001.pdf]

Electronic Supplementary Material (ESI) for RSC Advances.
This journal is © The Royal Society of Chemistry 2022

A-site deficient semiconductor electrolyte $Sr_{1-x}CO_xFeO_{3-\delta}$ for low-temperature (450-550 ^oC) solid oxide fuel cell

Yuzheng Lu[1], M.A.K. Yousaf Shah[2*], Naveed Mushtaq[2], Muhammad Yousaf[2], Muhammad Imran Asghar*[3,4], Peter D. Lund[3], Bin Zhu[2]

[1]*School of Electronic Engineering, Nanjing Xiao Zhuang University, 211171 Nanjing, China.*

[2]*Jiangsu Provincial Key Laboratory of Solar Energy Science and Technology/Energy Storage joint Research Center, School of Energy and Environment, Southeast University, No.2 Si Pai Lou, Nanjing 210096, China.*

[3]*New Energy Technologies Group, Department of Applied Physics, Aalto University School of Science, P. O. Box 15100, FI-00076 Aalto, Espoo, Finland*

[4]*Faculty of Physics and Electronic Science, Hubei University, Wuhan, Hubei, 430062, China.*

Corresponding author, MAK Yousaf Shah

Email address: alikamranshah91@gmail.com

Corresponding author, Muhammad Imran Asghar

Email address: imran.asghar@aalto.fi

Fig.1(a) shows the SEM image of Ni-foam revealing the fine porous structure suggesting that Ni-foam is contributing significant role in the higher catalytic activity of fuel cell device. Also, Image J software was used to determine the pore size of Ni foam where different pore have different size, but the mean size was 112.8 um as shown in Fig.1(b). Also, Fig.1(c) shows the cross-sectional view of the pellet of SCF electrolyte after testing and Ni-NCAL symmetrical electrodes revealing the electrolyte is well sandwiched between two symmetrical porous electrodes.

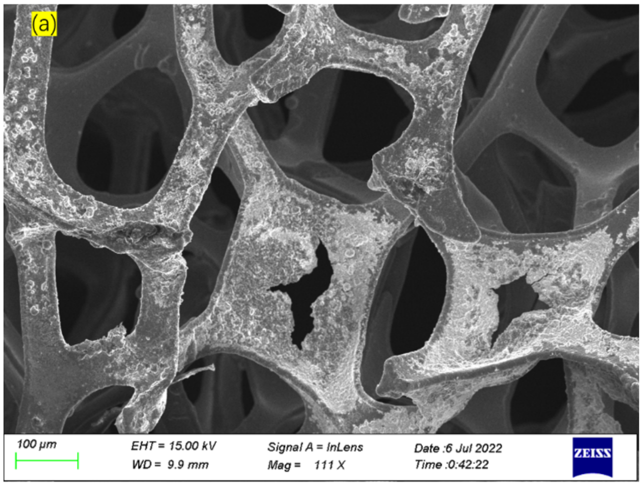

File Edit Font Results

(b)	Label	Area	Mean	Min	Max	Angle	Length
1		84.828	63.430	40.584	142.509	4.379	137.015
2		69.680	37.397	20.641	143.139	-23.199	112.479
3		108.308	62.080	39.320	185.652	-41.583	175.239
4		55.669	41.251	21.468	232.000	-5.906	89.707
5		77.254	60.199	22.386	206.452	-34.552	124.779
6		49.609	47.443	21.137	202.000	-12.433	80.031
7		43.550	39.592	21.477	208.867	38.972	70.447
8	Mean	69.843	50.199	26.716	188.660	-10.617	112.814
9	SD	22.586	11.404	9.064	34.138	27.079	36.582
10	Min	43.550	37.397	20.641	142.509	-41.583	70.447
11	Max	108.308	63.430	40.584	232.000	38.972	175.239

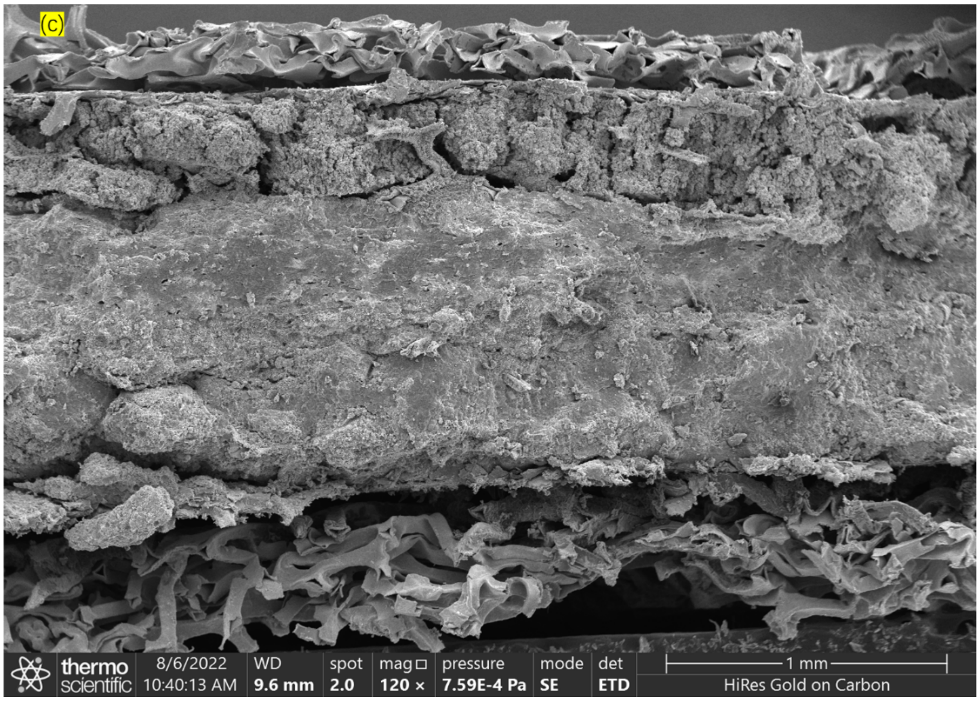

Fig. 1(a-c) Ni-foam and pore size of Ni-foam and SEM cross-sectional view of the prepared pellet of SCF electrolyte and Ni-NCAL electrodes

XPS spectra of sr-3d, Co-2p and Fe-2p have been investigated and presented in the Fig.2(a-c).

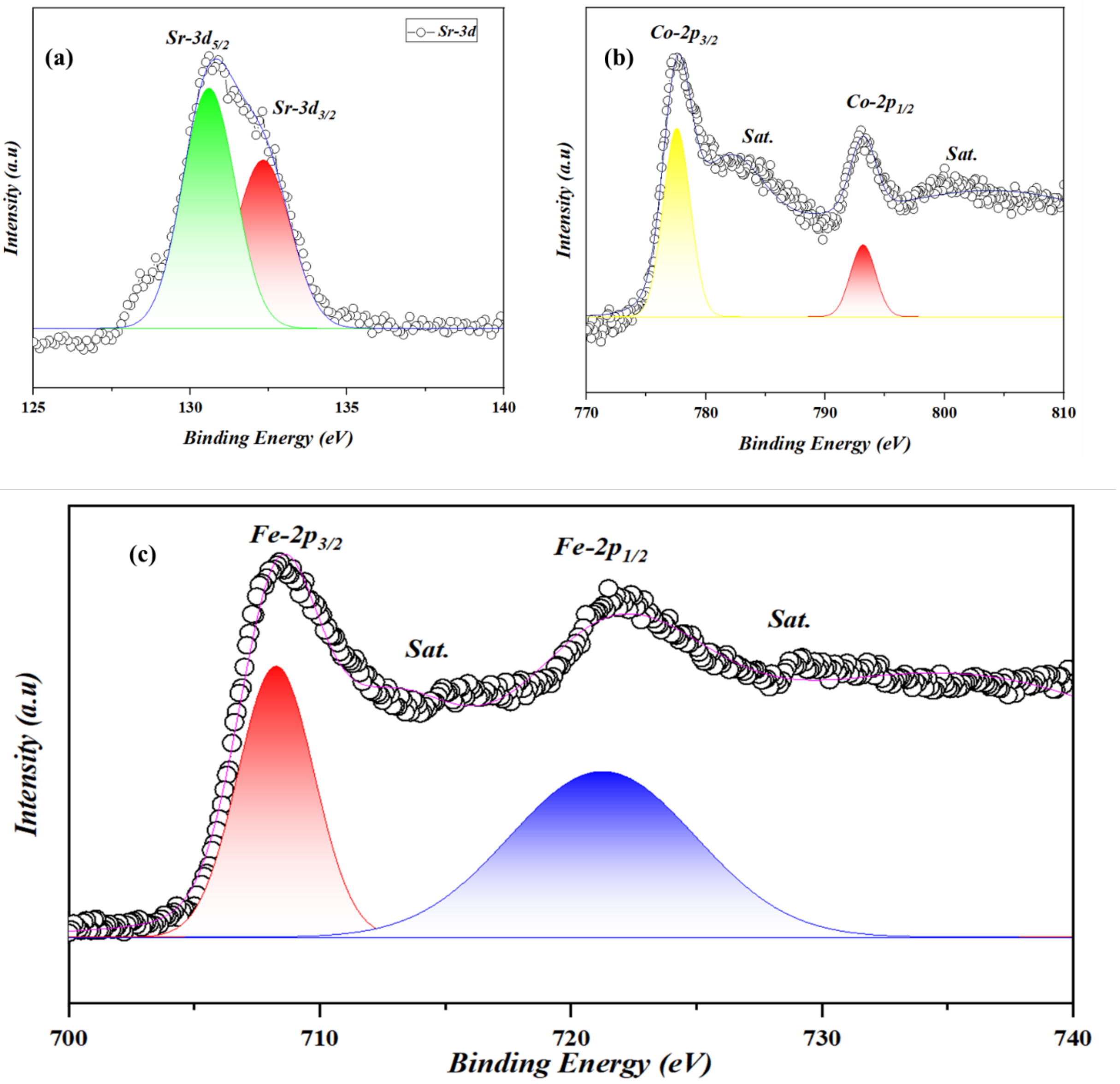

Fig.2(a-c) XPS spectra of Sr-3d, Co-2p and Fe-2p

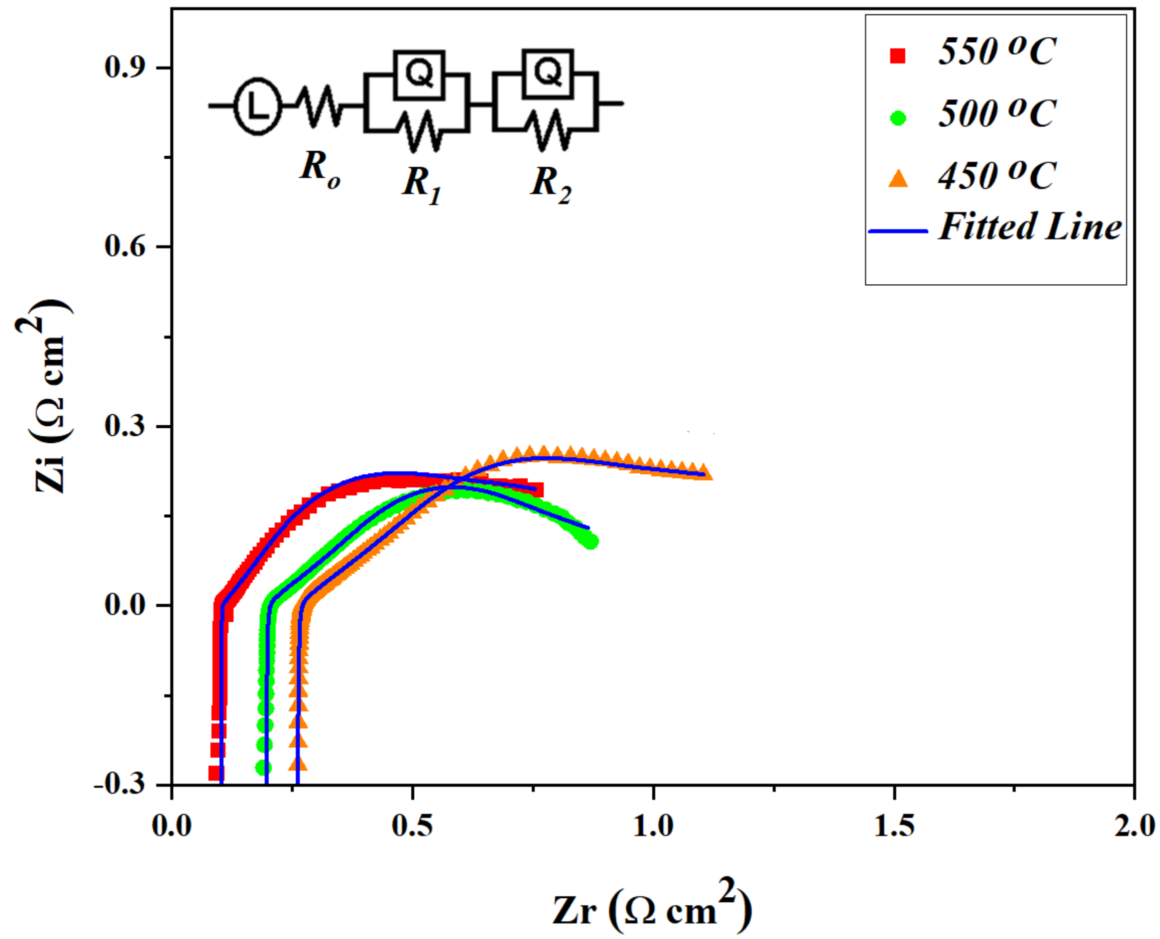

Fig.3 EIS spectra of $SrCo_{0.3}FeO_3$ electrolyte under H_2/Air environment at 550-450°C
